# A novel pathway for outer membrane protein biogenesis in Gram‐negative bacteria

**DOI:** 10.1111/mmi.13082

**Published:** 2015-07-17

**Authors:** Mark Jeeves, Timothy J. Knowles

**Affiliations:** ^1^ School of Cancer SciencesUniversity of BirminghamBirminghamB15 2TTUK

## Abstract

The understanding of the biogenesis of the outer membrane of Gram‐negative bacteria is of critical importance due to the emergence of bacteria that are becoming resistant to available antibiotics. A problem that is most serious for Gram‐negative bacteria, with essentially few antibiotics under development or likely to be available for clinical use in the near future. The understanding of the Gram‐negative bacterial outer membrane is therefore critical to developing new antimicrobial agents, as this membrane makes direct contact with the external milieu, and the proteins present within this membrane are the instruments of microbial warfare, playing key roles in microbial pathogenesis, virulence and multidrug resistance. To date, a single outer membrane complex has been identified as essential for the folding and insertion of proteins into the outer membrane, this is the β‐barrel assembly machine (BAM) complex, which in some cases is supplemented by the Translocation and Assembly Module (TAM). In this issue of *Molecular Microbiology*, Dunstan *et al*. have identified a novel pathway for the insertion of a subset of integral membrane proteins into the Gram‐negative outer membrane that is independent of the BAM complex and TAM.

## Introduction

The Gram‐negative bacterial membrane is highly complex and consists of a double membrane separated by a periplasmic space which acts as a permeability barrier that protects against environmental stresses, for example the presence of antibiotics or the harsh environment of the stomach. The outer of these two membranes, dubbed the outer membrane (OM), makes direct contact with the external environment and is essential for bacterial cell viability. The proteins in this membrane are vital in the maintenance of cellular homeostasis, allowing the excretion of toxic substances, such as antibiotics, and the uptake of nutrients. OM proteins are also the instruments of microbial warfare playing key roles in microbial pathogenesis, virulence and multidrug resistance as well as mediating many of the lethal processes responsible for infection and disease progression. Traditionally, two classes of protein have been recognised within the OM, peripheral membrane proteins, tethered to the membrane by virtue of an N‐terminal acyl group, and integral membrane proteins, characterised by their unique β‐barrel fold architecture. These β‐barrel membrane proteins are found exclusively in the OMs of Gram‐negative bacteria and eukaryotic organelles of prokaryotic origin, mitochondria and chloroplasts.

**Figure 1:**
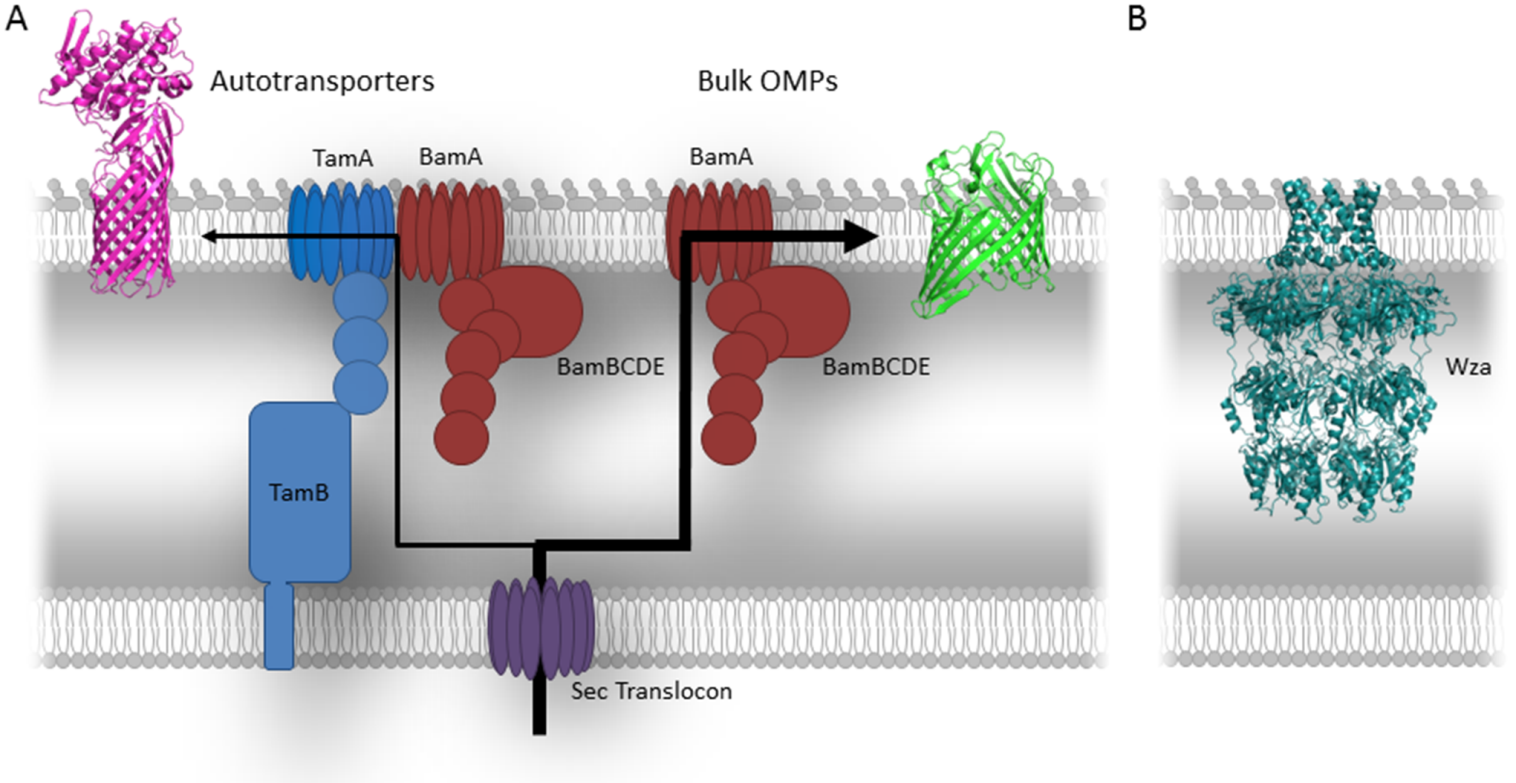
Current known pathways for outer membrane protein folding in Gram‐negative bacteria. A. All OMPs are synthesised in the cytoplasm and then targeted to the outer membrane via the SecYEG translocon. On entering the periplasm the majority of OMPs are targeted for folding via the Bam complex; however a subset of OMPs, the autotransporters, have been shown to require, in addition to the Bam complex, the translocation and assembly module or TAM. The exact role TAM plays remains unknown, but it is currently believed to provide another protein:lipid interface, analogous to that provided by BamA. Exactly why autotransporters require this complex is not clear, but presumably it is due to their more complex domain structure, e.g. β‐domain and passenger domain. B. Wza, an example of a secretion pore, is responsible for the transport of capsular polysaccharide across the outer membrane, which does not conform to the classical β‐barrel topology of other outer membrane proteins.

All OM proteins are synthesised in the cytoplasm as precursors, with an N‐terminal signal sequence that targets them to the SEC machinery which then transports them across the inner membrane. On entering the periplasm, the signal sequence is cleaved and the precursor OMPs escorted through the aqueous environment of the periplasm by chaperone proteins that maintain the OMP in a partially unfolded state. When the nascent OMP arrives at the OM, the prevailing view is that OMPs assemble into their characteristic β‐barrel fold structure and insert into the membrane with the help of the β‐barrel assembly machinery (BAM) complex (Knowles *et al*., 2009) and in some cases the translocation and assembly module (TAM) (Selkrig *et al*., 2014) (Fig. 1A). However, a subset of OMPs has been identified that do not conform to the classical β‐barrel fold topology (Fig. 1B). The secretion pores are a diverse set of proteins that are composed of oligomers with either α‐helical or β‐stand transmembrane segments. The assembly of these various oligomeric pores therefore represents an enigma, as the only known mechanism to insert proteins into the OM is either by the BAM complex or BAM complex and TAM. However, these complexes are only known to catalyse the sequential integration of β‐strands of those proteins with β‐barrel topology (Noinaj *et al*., 2013).

In this issue of *Molecular Microbiology*, Dunstan *et al*. (2015) propose a model for a second pathway for the insertion of integral membrane proteins into the Gram‐negative bacterial OM that is independent of both the BAM complex and TAM accounting for those proteins that do not adhere to the classical β‐barrel topology. The authors focused on three diverse oligomeric pores: (i) Wza, responsible for the secretion of capsular polysaccharides, whose transmembrane domain is composed of eight α‐helices (Dong *et al*., 2006); (2) GspD, a prototypical secretin protein that forms a homo‐dodecamer of ∼ 900 KDa, anchored integrally by a C‐terminal segment of peptide to form an α helical membrane pore (Dunstan *et al*., 2013), and (3) CsgG, of the curli translocon that is essential in the formation of biofilms, which forms a 36 β‐strand transmembrane domain, with four β‐strands contributed by each of the nine CsgG subunits (Goyal *et al*., 2014). By placing the desired pore gene under the control of an anhydro‐tetracycline (AhT) inducible promoter, together with a C‐terminal tetra‐cysteine (F1AsH) tag, they could control expression by the addition of AhT and simply observe membrane insertion and pore formation by SDS‐PAGE and visualisation by Lumio Green.

In an elegant experiment using *Escherichia coli* strain MC41000A, where BamA and TAM expression levels can be controlled by growth on either arabinose (expression) or glucose (depletion), conditions could be modulated whereby the capsular pore protein could be expressed by the presence of AhT while BamA levels could be depleted by growth in glucose. The authors observed that under depletion conditions in which the presence of the BAM complex or TAM can no longer be detected but cell death has not yet occurred, expression of Wza, CsgG or GspD could be induced and their appearance in the OM detected, whereas levels of the major OM proteins OmpC and OmpA were diminished by at least 95%, suggesting that the insertion of the capsular pores into the OM is independent of the BAM complex or TAM. The authors go on to propose a model for how these non‐standard OMPs may be transported and integrated into the OM.

## Membrane targeting

It has been observed previously that secretins, such as GspD and PulD, have associated pilotin molecules, small lipoproteins that recognise a targeting signal (S‐region) at the C‐terminus of the monomeric secretin (Koo *et al*., 2012). The pilotins then facilitate oligomerisation, insertion and proper assembly into the bacterial OM (Gu *et al*., 2012). The proposition here is that these pilotins, by virtue of their lipoprotein nature, may also act to target the nascent OMPs to the OM (Dunstan *et al*., 2015). The LOL machinery engages with lipoproteins on entering the periplasm and targets them for deposition at the OM, in the case of the pilotin molecule; however, it is proposed that the bound secretin is also transported to the OM. This is consistent with studies that have shown that in the absence of its pilotin molecule (PulS), PulD mislocates to the inner membrane (Guilvout *et al*., 2006).

In other cases, the secretins themselves have been discovered to be lipoproteins and have dispensed with the pilotin in the targeting phase of the pathway (Viarre *et al*., 2009). This also seems to be the case with the capsular pores formed by CsgG and Wza, both are lipoproteins, and mutagenesis of the lipoprotein targeting sequence leads to the proteins being targeted to the periplasm rather than the membrane, this strongly suggests that the LOL pathway is responsible for membrane targeting in these cases as well (Nesper *et al*., 2003; Goyal *et al*., 2014).

## Insertion

**Figure 2:**
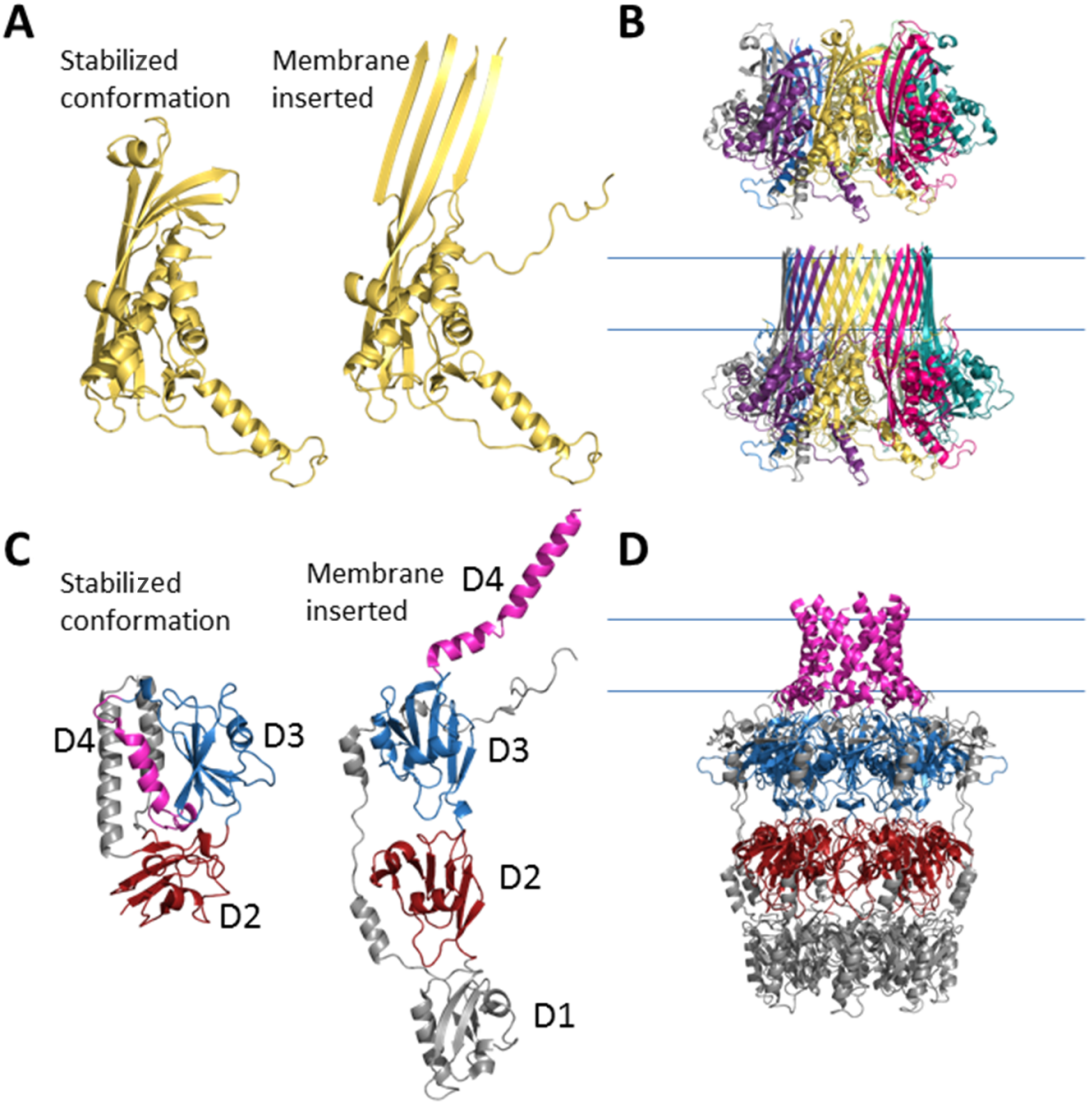
Capsular pore forming proteins can adopt an aqueous stabilised pre‐integration form and a membrane inserted pore conformation. A. Crystal structure of a single subunit of CsgG in both its aqueous stabilised and membrane inserted form showing the change in position of a single amphipathic segment that forms part of the pore and (B) the representative structures shown in their respective complexes. C. Monomeric crystal structures of homologous proteins GfcC and Wza in an aqueous stabilised conformation (GfcC) and membrane inserted conformation (Wza) showing the presence of the carboxyl‐terminal amphipathic helix (D4) either stabilised against D3, one of two tandem grasp domains (D2 and D3) present or elongated in a membrane inserted α‐helical pore conformation as part of a complex of eight monomers shown in (D).

On arrival at the OM, how do the proteins insert into the membrane? Dunstan *et al*. believe the answer lies in the observation of variant crystal structures observed for these capsular pores. In the case of CsgG, a membrane‐integrated form exists in which the β‐strands from each of the nine monomers form a transmembrane domain. The structure of an aqueous pre‐integration form of CsgG in which the β‐stands that will form the transmembrane domain are not exposed has also been solved (Fig. 2A and B). This pre‐integration form consists of a complex of eight monomers of CsgG, and the mechanism by which this is converted to the nine monomers in the membrane integrated form is not understood. A similar mechanism may be also be responsible for the correct membrane insertion of Wza (Sathiyamoorthy *et al*., 2011). Comparisons between the structures of Wza and the homologous GfcC also show two different conformations (Fig. 2C and D). In the case of GfcC and Wza, these pores are alpha helical in nature, and the crystal structure of GfcC shows the C‐terminal α‐helix packed against the protein in a conformation that would preclude its integration into the OM. However, it has not yet been established whether GfcC forms a capsular pore in this peripheral membrane protein conformation, or if conformation changes release the C‐terminal helix ready for membrane integration. However, the striking similarities between the structural forms of Wza and GgcC are inescapable.

The authors therefore propose a model by which the monomeric forms of secretins and capsular pore proteins adopt an aqueous stabilised pre‐integration form on entering the periplasm, in some cases this may be aided by pilotins, which shield the presumptive transmembrane segments, as has been observed for PulD (Hardie *et al*., 1996; Collin *et al*., 2011). The recognition of a pilotin, or the protein subunit itself, by the Lol machinery, results in the targeting of the secretion pore monomers to the OM where homo‐oligomerisation occurs. It has been proposed that, during this homo‐oligomerisation reaction, a conformational change occurs that drives integration of an amphipathic segment, four β‐stands in each subunit for CsgG and a C‐terminal α‐helix in the case of Wza, into the OM where they form a pore through the membrane (Huysmans *et al*., 2013; Goyal *et al*., 2014; Guilvout *et al*., 2014). Presumably during this event any attached pilotin is released. At present it is not known whether this homo‐oligomerisation is driven purely by concentration, or whether there is also a significant entropic component derived from the large buried surface formed from the monomer–monomer contacts (Cao *et al*., 2014). The possibility that there is an unknown assembly machinery that coordinates the reaction and thereby catalyses the assembly of these diverse secretion pores cannot be completely excluded.

The protein subunits of these secretion pores belong to diverse protein families, and the segments of protein traversing the OM are of distinct secondary structure but despite the structural diversity the authors propose a general mechanism for the assembly for all of these secretion pores into the OM of Gram‐negative bacteria (Dunstan *et al*., 2015), though whether this is true for all such proteins remains to be seen.

It is increasingly apparent that there are multiple pathways involved in the formation of the OM of Gram‐negative bacteria, highlighting the importance of this barrier. Differences in membrane spanning topologies require novel integration systems vital for correct function of the OM. Each one of these systems is potentially a target for antimicrobial agents. The way is now open for further studies into the assembly of these complex oligomeric pores and such studies in the future may give rise to novel classes of antibiotic.

## References

[mmi13082-bib-0001] Cao, B. , Zhao, Y. , Kou, Y. , Ni, D. , Zhang, X.C. , and Huang, Y. (2014) Structure of the nonameric bacterial amyloid secretion channel. Proc Natl Acad Sci USA 111: E5439–E5444. 25453093 10.1073/pnas.1411942111 PMC4273326

[mmi13082-bib-0002] Collin, S. , Guilvout, I. , Nickerson, N.N. , and Pugsley, A.P. (2011) Sorting of an integral outer membrane protein via the lipoprotein‐specific Lol pathway and a dedicated lipoprotein pilotin. Mol Microbiol 80: 655–665. 21338419 10.1111/j.1365-2958.2011.07596.x

[mmi13082-bib-0003] Dong, C. , Beis, K. , Nesper, J. , Brunkan‐Lamontagne, A.L. , Clarke, B.R. , Whitfield, C. , and Naismith, J.H. (2006) Wza the translocon for *E. coli* capsular polysaccharides defines a new class of membrane protein. Nature 444: 226–229. 17086202 10.1038/nature05267 PMC3315050

[mmi13082-bib-0004] Dunstan, R.A. , Heinz, E. , Wijeyewickrema, L.C. , Pike, R.N. , Purcell, A.W. , Evans, T.J. , *et al* (2013) Assembly of the type II secretion system such as found in *Vibrio cholerae* depends on the novel Pilotin AspS. PLoS Pathog 9: e1003117. 23326233 10.1371/journal.ppat.1003117 PMC3542185

[mmi13082-bib-0005] Dunstan, R.A. , Hay, I.D. , Wilksch, J.J. , Schittenhelm, R.B. , Purcell, A.W. , Clarke, J. , *et al* (2015) Assembly of the secretion pores GspD, Wza and CsgG into bacterial outer membranes does not require the Omp85 proteins BamA or TamA. Mol Microbiol 97: 616–629. 25976323 10.1111/mmi.13055

[mmi13082-bib-0006] Goyal, P. , Krasteva, P.V. , Van Gerven, N. , Gubellini, F. , Van den Broeck, I. , Troupiotis‐Tsailaki, A. , *et al* (2014) Structural and mechanistic insights into the bacterial amyloid secretion channel CsgG. Nature 516: 250–253. 25219853 10.1038/nature13768 PMC4268158

[mmi13082-bib-0007] Gu, S. , Rehman, S. , Wang, X. , Shevchik, V.E. , and Pickersgill, R.W. (2012) Structural and functional insights into the pilotin‐secretin complex of the type II secretion system. PLoS Pathog 8: e1002531. 22346756 10.1371/journal.ppat.1002531 PMC3276575

[mmi13082-bib-0008] Guilvout, I. , Chami, M. , Engel, A. , Pugsley, A.P. , and Bayan, N. (2006) Bacterial outer membrane secretin PulD assembles and inserts into the inner membrane in the absence of its pilotin. EMBO J 25: 5241–5249. 17082772 10.1038/sj.emboj.7601402 PMC1636608

[mmi13082-bib-0009] Guilvout, I. , Chami, M. , Disconzi, E. , Bayan, N. , Pugsley, A.P. , and Huysmans, G.H. (2014) Independent domain assembly in a trapped folding intermediate of multimeric outer membrane secretins. Structure 22: 582–589. 24657091 10.1016/j.str.2014.02.009

[mmi13082-bib-0010] Hardie, K.R. , Seydel, A. , Guilvout, I. , and Pugsley, A.P. (1996) The secretin‐specific, chaperone‐like protein of the general secretory pathway: separation of proteolytic protection and piloting functions. Mol Microbiol 22: 967–976. 8971717 10.1046/j.1365-2958.1996.01539.x

[mmi13082-bib-0011] Huysmans, G.H. , Guilvout, I. , and Pugsley, A.P. (2013) Sequential steps in the assembly of the multimeric outer membrane secretin PulD. J Biol Chem 288: 30700–30707. 24019525 10.1074/jbc.M113.489112 PMC3798540

[mmi13082-bib-0012] Knowles, T.J. , Scott‐Tucker, A. , Overduin, M. , and Henderson, I.R. (2009) Membrane protein architects: the role of the BAM complex in outer membrane protein assembly. Nat Rev Microbiol 7: 206–214. 19182809 10.1038/nrmicro2069

[mmi13082-bib-0013] Koo, J. , Burrows, L.L. , and Howell, P.L. (2012) Decoding the roles of pilotins and accessory proteins in secretin escort services. FEMS Microbiol Lett 328: 1–12. 22098485 10.1111/j.1574-6968.2011.02464.x

[mmi13082-bib-0014] Nesper, J. , Hill, C.M. , Paiment, A. , Harauz, G. , Beis, K. , Naismith, J.H. , and Whitfield, C. (2003) Translocation of group 1 capsular polysaccharide in *Escherichia coli* serotype K30. Structural and functional analysis of the outer membrane lipoprotein Wza. J Biol Chem 278: 49763–49772. 14522970 10.1074/jbc.M308775200

[mmi13082-bib-0015] Noinaj, N. , Kuszak, A.J. , Gumbart, J.C. , Lukacik, P. , Chang, H. , Easley, N.C. , *et al* (2013) Structural insight into the biogenesis of beta‐barrel membrane proteins. Nature 501: 385–390. 23995689 10.1038/nature12521 PMC3779476

[mmi13082-bib-0016] Sathiyamoorthy, K. , Mills, E. , Franzmann, T.M. , Rosenshine, I. , and Saper, M.A. (2011) The crystal structure of *Escherichia coli* group 4 capsule protein GfcC reveals a domain organization resembling that of Wza. Biochemistry 50: 5465–5476. 21449614 10.1021/bi101869h

[mmi13082-bib-0017] Selkrig, J. , Leyton, D.L. , Webb, C.T. , and Lithgow, T. (2014) Assembly of beta‐barrel proteins into bacterial outer membranes. Biochim Biophys Acta 1843: 1542–1550. 24135059 10.1016/j.bbamcr.2013.10.009

[mmi13082-bib-0018] Viarre, V. , Cascales, E. , Ball, G. , Michel, G.P. , Filloux, A. , and Voulhoux, R. (2009) HxcQ liposecretin is self‐piloted to the outer membrane by its N‐terminal lipid anchor. J Biol Chem 284: 33815–33823. 19815547 10.1074/jbc.M109.065938 PMC2797151

